# Advocacy – defending science or destroying it? Interviews with 47 climate scientists about their fundamental concerns

**DOI:** 10.1177/09636625251314164

**Published:** 2025-02-03

**Authors:** Lydia Messling, Yuyao Lu, Christel W. van Eck

**Affiliations:** Lydia Messling Consulting, UK; University of Amsterdam, The Netherlands

**Keywords:** climate advocacy, climate change, climate scientists, communication ethics, interviews, philosophy of science, policy advocacy, science communication, value-free ideal

## Abstract

The discourse on scientists’ involvement in climate advocacy has intensified, with a growing number participating in civil disobedience. This trend has sparked criticism within the academic community. We conducted 47 interviews with climate scientists about the fundamental concerns that underpin their arguments. Scientists worry that advocacy may compromise scientific impartiality and invite allegations of biased science and abuse of authority. Despite this, some scientists view informing and warning the public as their duty and as an act of defending science’s credibility. Concerns about independence and the role of scientists in society exist at both ends of the debate, underscoring the challenging landscape scientists currently navigate. While this article does not comment on the acceptability of advocacy, we propose that scientists engage in discussions about their duties and delineate the types of values deemed acceptable for incorporation in science communication about climate change.

## 1. Introduction

Climate change poses irreversible consequences (IPCC, 2023). Within this crisis, the role of climate scientists in advocacy has become increasingly salient. Notable examples include their participation in petitions and protests advocating for the acceleration of climate mitigation (Capstick et al., 2022; Tollefson, 2021). The highly politicized and polarized nature of the ‘climate change debate’ (Bolsen and Shapiro, 2017) spurs questions about how climate scientists can publicly endorse climate action or specific climate policies.

Many supporters of climate advocacy underscore the role of civil disobedience in driving transformative social change (Gardner and Wordley, 2019). They argue that scientists engaging in advocacy is justified as they regard it as an essential social responsibility of scientists in times of crisis (Capstick et al., 2022; Thompson, 2020). However, concerns have been raised regarding the potential conflict between advocacy and the pursuit of objective, value-free science (Nelson and Vucetich, 2009). It is argued that engaging in policy advocacy may lead to the perception that scientific knowledge is illegitimately influenced by political values or that experts are abusing their trusted positions to further political agendas. Consequently, this perception threatens to undermine scientific credibility and objectivity (Nelson and Vucetich, 2009; Sharman and Howarth, 2017; Tollefson, 2021).

Contrary to the large public support for scientists engaging in climate advocacy (Cologna et al., 2021), previous research indicates that scientists themselves have mixed views. Commentary papers reflect varying personal views about scientists engaging in advocacy (e.g. Büntgen, 2024; Capstick et al., 2022; Green, 2020; Tollefson, 2021; van Eck et al., 2024). Other theoretical papers aim to conceptualize scientists’ engagement in advocacy and what scientists’ role is in society (e.g. Donner, 2014; Nelson and Vucetich, 2009; Pielke, 2007). Survey studies among climate scientists show that scientists feel that they should publicly and politically engage, but there are mixed views about climate advocacy (Boykoff and Oonk, 2020; Cologna et al., 2021; Dablander et al., 2024). Other surveys tested the claim that advocacy hurts climate scientists’ credibility, and found that advocacy did not inherently undermine their credibility with the general public (e.g. Cologna et al., 2022; Kotcher et al., 2017).

While these studies are valuable for identifying the general views of the academic community, they tend to miss the complex, multidimensional views on climate advocacy, reducing scientists’ involvement in climate advocacy to a binary action. To name a few examples, the expertise of the scientist (e.g. Sharman and Howarth, 2017), the cause one advocates for (e.g. Kotcher et al., 2017), scientists’ level of seniority (e.g. Boykoff and Oonk, 2020; van Eck, 2023), the role of uncertainties in the presentation of scientific evidence (e.g. Donner, 2014), the use of academic symbols and the type of activities (e.g. op-ed in newspaper versus civil disobedience) are all factors that play a role in the perception of advocacy engagement.

This underscores the necessity for qualitative research that captures the complexity and nuance of climate scientists’ views on advocacy. While previous studies have identified some concerns about advocacy among climate scientists (Boykoff and Oonk, 2020; Sharman and Howarth, 2017; van Eck, 2023), more fundamental questions remain underexplored, such as why scientists hold these concerns and what roles they believe scientists should play in society. Specifically, our study aims to explore two critical dimensions: epistemological concerns related to the integrity, objectivity and credibility of scientific knowledge, and ontological concerns regarding the roles and responsibilities of scientists within a societal context.

Understanding the reasoning behind scientists’ attitudes towards climate advocacy is crucial for fostering a more nuanced, accurate, and productive discourse within the scientific community. Without clarity on the fundamental concerns about climate advocacy, there is a risk of miscommunication or ‘talking past each other’. To address this gap, we explore why scientists view advocacy as either a potential threat or non-threat through in-depth interviews with 47 climate scientists. Without making recommendations about what constitutes acceptable or unacceptable advocacy, we aim to map the fundamental epistemological and ontological concerns that climate scientists hold regarding advocacy.

## 2. Literature review

### Climate scientists in public debates

Scientists face numerous (perceived) challenges in their public engagement efforts, including common barriers such as a lack of time and formal training; negotiating a polarized society characterized by misinformation and climate scepticism; deciding which messages to communicate and how to frame them; and navigating the debate about how scientists can engage in climate advocacy (van Eck, 2023).

Previous research finds that scientists hold a strong sense of duty to communicate their findings to the public and policymakers (Sharman and Howarth, 2017; van Eck, 2023). There has been a recent surge in calls for climate scientists to shift from publication-focused activities towards direct public actions that can influence policy and societal responses to climate change (Capstick et al., 2022; Gardner and Wordley, 2019; Green, 2020). Research found that climate scientists are more likely to engage in public debate when it is perceived as positive and within their control, and their willingness often increases when they observe their peers participating in similar activities (Poliakoff and Webb, 2007).

However, though the terms are often used interchangeably, it is crucial to distinguish between participation in public debates and climate advocacy. In our context, we define advocacy as “a plea in active support of something, with the intent to persuade others to believe and act similarly”. Public engagement or science communication does not plea but aims to relay information to the public and policymakers about research processes and results. However, as we shall explore later on in this article, it can sometimes be difficult to make this distinction in practice.

### Value judgements and climate advocacy

Many philosophers of science assert that science cannot be separated from values, and therefore the call for value-free science is not possible and indeed, not desirable. What we determine to be ‘good science’ is shaped by value judgements. Values are part of how science operates; they are (legitimately) involved in scientific judgements made by scientists themselves, as well as in the understanding and assessment of the role that science is to play in society (Douglas, 2009; Nagel, 1961). The discourse of values in science is relevant in the context of advocacy, as scientists engaging in advocacy apply additional value judgements in how and what it is they advocate, such as their political worldview.

The concern is that the political values associated with advocacy may then also exert an unacceptable influence upon the creation of scientific knowledge, resulting in biased science or the ‘Stealth Advocacy’ that Pielke (2007) describes. However, just engaging in policy advocacy does not automatically mean that political values have unacceptably influenced the creation of scientific knowledge. The mechanisms of peer-review and methodological design (while their effectiveness can vary) are designed in part to filter out biased science. Therefore, the threat that advocacy poses to the integrity of science is nuanced.

Climate change represents a ‘post-normal situation’ characterized by high uncertainties and significant decision stakes, where stand-alone scientific inputs are insufficient, and the value judgments of other actors must also be considered (Funtowicz and Ravetz, 1993). This form of post-normal science is defined by Brüggemann et al. (2020: 9) as involving ‘scientific roles, norms, and practices that emerge from post-normal situations and diverge from established norms of science’. There is no clear or distinct boundary between science and non-science (e.g. policy, activism) (Gieryn, 1983), and post-normal science further blurs the boundaries between science and society (Brüggemann et al., 2020). Consequently, post-normal science communication differs from traditional practices by actively engaging with the public and addressing the uncertainties and value-laden questions surrounding their research. Brüggemann et al. (2020: 11) argue that ‘future research should investigate more closely what type of advocacy scientists [. . .] support or reject’.

Research and university ethical committees have sought to categorize climate advocacy, ranging from simple binary classifications to complex models assessing advocacy based on value judgments. Donner’s (2014) ‘science-advocacy continuum’ is a spectrum from objective judgments to normative judgments. The degree of advocacy increases when factors such as worldviews, scientific uncertainty, and professional risk exert more influence. For instance, advocating for the existence of anthropogenic climate change based on robust scientific evidence is at the other end to advocating for specific policy interventions based on economic preferences, where normative judgments play a more prominent role.

When communicating their work, scientists inherently select which messages to convey and how to frame them for different audiences. For example, they decide whether to emphasize scientific consensus (e.g. van der Linden et al., 2015) or highlight uncertainties in their research (e.g. van Eck, 2023). Another example includes whether it is acceptable for scientists to use the ‘climate emergency’ frame (Markusson et al., 2014; Ripple et al., 2020). In addition, they must consider how to frame policy options, either as imperatives or as options (Post and Bienzeisler, 2024).

Generally, climate scientists have mixed views on engaging in climate advocacy, with reluctance often stemming from fears of losing credibility, criticizing colleagues, and impacting their academic careers (Boykoff and Oonk, 2020; van Eck, 2023). Other research highlights that, while opinions on advocacy for policy directions vary, there is broad support within the scientific community for evidence-based advocacy (Boykoff and Oonk, 2020; Cologna et al., 2021). Schmidt (2015) argues that all climate communication involves some form of advocacy, whether for deepening public understanding, raising awareness, or promoting specific policies. He suggests that the debate should shift from whether scientists should engage in advocacy to what they advocate for.

### Credibility, objectivity and trust

Scientists hold trusted positions in society (Cologna et al., 2021), and credibility, objectivity, and trust (Besley et al., 2021) are pivotal in maintaining their boundary arrangements. Credibility, defined as the extent to which scientists’ assertions are esteemed by the lay audience (Barnes, 2005), is intricately linked with the type of objective knowledge the scientist creates (Douglas, 2009). Crucially, assessing objectivity via the scientific method is done (and can only be done) by scientists’ peers. To this extent then, credibility is also related to the scientific community’s opinion of the scientist’s contribution to ‘good objective knowledge’. The concern about advocacy threatening credibility is therefore twofold: that it could undermine the credibility of science with the public, and/or that it could threaten the credibility of the scientist(s) with their community questioning their ability to create objective knowledge.

Contrary to concerns, there is evidence that suggests that climate advocacy does not detrimentally impact public perceptions of climate scientists’ credibility (Cologna et al., 2021; Kotcher et al., 2017). Kotcher et al. (2017) showed that climate scientists can engage in policy advocacy without undermining their perceived credibility with the public, particularly when advocating for widely accepted measures like reducing carbon dioxide emissions. However, advocacy for certain actions, such as the construction of nuclear power plants, may pose exceptions, potentially influencing scientists’ perceived credibility. Similarly, Cologna et al. (2021) demonstrated that advocacy does not compromise public perceptions of scientists’ credibility and trustworthiness, although it may have nuanced impacts on perceived objectivity. Post and Bienzeisler (2024) contribute to this discussion by proposing that climate scientists can enhance public trust by adopting the role of ‘Honest Brokers’; when scientists present policy advice as options rather than imperatives, they have the potential to garner increased public trust.

These first studies suggest that there is substantial public support for scientists to advocate for climate policies, with evidence suggesting that the public may even anticipate greater advocacy from scientists than scientists themselves are comfortable with, particularly in contexts like the United States.

## 3. Methodology

### Research design

We conducted 47 in-depth interviews with climate scientists to gain a deep understanding of their views on climate advocacy and to explore their fundamental concerns through follow-up questions (Gubrium and Holstein, 2012). Data analysis followed the principles of the grounded theory approach (Charmaz, 2006). The research received formal approval from the Ethics Review Board of the Politics Department at the Univeristy of Reading, UK.

### Data collection

With both a purposive and snowball sampling design, 60 climate scientists were approached by email, achieving a response rate over 90%. The final sample consisted of 47 climate scientists (see Supplement) based in the United States (27) and the United Kingdom (20). These countries were selected as the United States is characterized by high climate change polarization (Bolsen and Shapiro, 2017), whereas climate change is generally more accepted in the United Kingdom (Fisher et al., 2018).

All 47 interviewees are recognized as experts in climate change. Of these, 41 identified as natural scientists directly conducting climate research. The remaining six were primarily involved in the communication of specifically climate science, assisting natural climate scientists in their communications. They were included because they have significant experience with the different tensions that scientists experience in their communications.

The interviewees were affiliated with a variety of organizations, including universities (e.g. University of East Anglia), research institutes (e.g. NASA Goddard Institute for Space Studies) and research centres (e.g. Tyndall Centre for Climate Change Research). In addition, some interviewees held dual affiliations, working with both academic/research institutions and other entities such as government agencies. The interviewer had no prior relationships with any interviewees before conducting the interviews.

Based on the literature and insights gained from eight unstructured pilot interviews with climate scientists affiliated with the meteorology department at the University of Reading, a topic guide was developed consisting of six themes: (1) Defining advocacy; (2) Acceptable advocacy; (3) Independence & credibility; (4) Rights & duties as a scientist/citizen; (5) Ethics of advocacy/framing; and (6) Practical methods. Each theme included keywords, aiding in the formulation of follow-up questions and functioning as verbal probes.

The in-depth interviews were conducted between April and August 2018, averaged a duration of 1 hour and 10 minutes, with 11 of them conducted via telephone/video call and 36 held face-to-face. All interviews were recorded with the consent of the interviewees. Data collection halted upon reaching data saturation.

### Data analysis

All interviews were transcribed in a full-verbatim manner and coded in NVivo software. Data analysis followed an iterative process guided by the grounded theory approach (Charmaz, 2006). First, with the themes of the interview guide in mind, transcripts were subjected to line-by-line coding. This inductive approach resulted in a list of 191 codes (open coding) (see Supplement for overview of codes and sample of the coded data). Subsequently, we conducted axial coding to identify different points of view on the subject of advocacy, including ‘Biased science’, ‘Abuse of authority’, ‘Defence of Science’ and ‘Moral obligation/right as a citizen’. Redundant codes were removed. Finally, selective coding was applied to refine the major concerns to address our research question more directly, identifying ‘Integrity of science’ and ‘Role of scientists in society’ as the overarching categories to explain interviewees’ views on whether they find advocacy a threat or if it is needed (see Supplement).

## 4. Results

Two main themes emerged in the arguments for and against engaging in climate policy advocacy: the integrity of science and the role of the scientist in society. Table 1 categorizes these arguments in a framework, with ‘A’ indicating arguments against advocacy and ‘F’ indicating arguments in favour. Interviewees expressed views about their fundamental concerns but without using the exact terminology used in this table. When provided with the language and framing of the categories of this table (though they were not shown the table framework), interviewees broadly agreed that the fundamental concerns accurately described what they perceive as ‘threatening’ about advocacy and why advocacy is ‘needed’. The theme of integrity of science relates to epistemological concerns about maintaining the objectivity, validity, and reliability of scientific knowledge. Separately, the role of scientists in society aligns with ontological concerns about what the roles and responsibilities of scientists should be.

**Table 1.** Categorization of concerns to avoid or engage in climate advocacy.

	Epistemological concerns	Ontological concerns
	*Integrity of science (1)*	*Role of scientists in society (2)*
*Advocacy threatens (A)*	Biased science (A1)	Abuse of authority/position (A2)
*Advocacy is needed (F)*	Defence of science (F1)	Duty as experts and citizens (F2)

‘A’ stands for ‘Against advocacy’ and ‘F’ for ‘For advocacy’

Advocacy was seen to threaten the integrity of science by the creation (or suspicion) of biased science (A1) – where political values illegitimately influence scientific knowledge, created for the purpose of supporting a particular political opinion. Similarly, engaging in advocacy on topics outside a scientist’s expertise was viewed as a misuse of their authority and position in society (A2). This ontological concern reflects worries about scientists overstepping their societal role and responsibilities.

At the same time, these concerns about preserving the integrity of science and the role of scientists were also central to arguments in support of advocacy. Interviewees emphasized that scientists should engage in advocacy when defending science and correcting misuses or misinterpretations of scientific evidence (F1). This defence of science aligns with how some interviewees viewed their duty as experts and citizens (F2), who are responsible for ‘sounding the alarm’ on critical issues like climate change.

However, this framework should not be viewed as a binary conceptual tool for distinguishing between arguments for and against advocacy. The fundamental concerns can coexist, making it challenging to map specific instances of advocacy onto this framework definitively. For example, an engagement might simultaneously address concerns against advocacy, such as accusations of biased science (A1), while being driven by the need to defend the science (F1). In addition, audience perception plays a crucial role in interpreting advocacy and can significantly influence whether an engagement is deemed acceptable or unacceptable. Different audiences may react differently to the same advocacy action, depending on factors such as the nature of the advocacy (e.g. civil disobedience), the status of the individuals involved (e.g. early career versus senior scientists), and the method of engagement (e.g. as individuals or as part of an organized group).

Thus, the framework clarifies the fundamental concerns about advocacy and their epistemological and ontological basis but should not be used to categorize specific advocacy actions. We will now explore these concerns and examples of uncertainty in more detail.

### Biased science (A1)

Engaging in climate advocacy was seen by some interviewees to potentially result in (perceived) biased science. Their concern was that policy advocacy might be seen as indicative of biased science, where research and results were contrived to support a particular political opinion:

“When you’re an advocate, in the activist sense, your passions are involved, and you’ve lost your objectivity. That doesn’t mean you can’t do valid science, but you’ve lost something in terms of the objectivity.”

Another way to bias science is when only one perspective is shared, failing to acknowledge uncertainty or dissenting viewpoints from others in the scientific community; presenting something as ‘certain fact’ to spur a particular course of action when really it is still unsettled. It was felt that even mere suspicion of biased science may have the potential to derail research and career, especially when this concern came from the scientific community. To that end, losing the trust and credibility of the scientific community is the primary concern as it is one’s peers that qualify and validate one’s research, credibility, and independence:

“You need a couple of things to make it as a scientist, at least initially, right, – and part of that is support. And if you lose the support from your community. . . And you’ll notice I haven’t said, oh, I’m fearing that I’m gonna lose other activity in my research. I don’t fear that.”

It was also suggested that scientists who raise concerns about biased science may only be doing so because they disagree with the (perceived) implications of scientific research done by others; accusation of biased science is used as a discourse tactic to discredit one’s opponent:

“It’s an ‘us versus them’ and you need to automatically discredit scientists that you perceive as [being] on the ‘them’ side . . . you’re discrediting the scientist, not critiquing their arguments.”

Similarly, others provided examples of past accusations received from outside of the scientific community accusing scientists of exaggerating or fabricating the narrative of climate change ‘conspiracy’ to make money:

“I mean a lot of the criticism I think the climate science community has come under has been along the lines of ‘you just wanna further your own careers and that’s why you’ve made up this, this climate change hoax, it’s all a load of nonsense, [. . .] you’ve erm cooked the books’.”

Seeking money, specifically advocating for research funding, was seen as acceptable and par for the course in scientific research, so long as it did not influence the outcomes of scientific research. However, some raised concerns about the possibility of tailoring research to align with the preferences of policymakers and funders for the sake of gaining funding or seeking other benefits such as enhancing their professional profiles, rather than focusing on making novel contributions to scientific knowledge. For example, while not necessarily accused of bias, attribution studies were criticized as being just for media attention:

“Attribution is basically associated with blame. So, if I talk about attribution, even climate signals like which for a scientist seems scientific, (. . .) then there’s a perception that you’re pushing for a certain solution that you’re advocating, that you have a vested interest or an agenda.”

On the contrary, attribution studies were described as helping to explain the impact of climate change to non-expert audiences. This is one example where trying to frame uncertainties for non-expert audiences may result in the perception that the scientist has engaged in advocacy via biased science when trying to simplify and explain.

#### Examples of perceived biased science in message and framing choices (A1 & A2)

How scientists strategically or unconsciously frame their messages is often a central element in potentially producing (the perception of) biased science (A1) or abusing their authoritative position in society (A2). Interviewees perceived that advocacy statements often include normative frames, denoted by words like ‘should’ and ‘ought’, as opposed to descriptions of ‘what is’ or hypotheses. Saying science ‘tells us what we should do’ could be described as trying to bias science:

“We define the advocacy based on a ‘should’, right? Not an ‘is’, and then it’s related to ‘oughts’, ‘should’. We can’t get ‘should’ from ‘is’. We’ve known that for a long time. So, anytime that you’re saying that there’s a normative statement about what people should know or should understand, or should do – all those things are advocacy.”

Other interviewees expressed concerns about the loss of nuance while simplifying messages, and thus being misinterpreted as making policy recommendations. They were aware that accurately communicating complex scientific concepts and risks to a non-expert audience necessarily involves making value judgements and therefore provides an opportunity for political values to influence a range of decisions. These include which frames are chosen to communicate through, how to simplify the science (which may lead to differences in opinion about what to leave out of a communication), and how to judge how the communication has been understood by the audience.

This last point in particular was raised several times: if a scientist makes a judgement about the audience’s comprehension based on their resulting actions, the scientist could appear to be biased depending on how they sought further engagement with that audience. For example, a scientist is seeking to engage a policymaking audience with new research which the scientist feels has implications for policymaking. The scientist tries to present their research objectively and is careful to not make any policy recommendations. The scientist thinks, however, that the research makes it obvious that *some* form of policy change is needed. When the scientist sees that no policy change has been implemented, they may think that the policymakers have not understood them correctly. The scientist then embarks upon further engagements with that audience to try and help them understand, but becomes frustrated when policy still does not align with what they think ‘is obvious from the science’. It could indeed be the case that the policymaker audience has not understood, or that they did fully understand but chose to take a different view on resulting policy action. Interviewees explained that this judgement by the scientist – that lack of action must be lack of understanding, not a difference in policy opinion – is based on political value judgements of what should happen next. Continued advocacy for ‘science-based policy’ could therefore be interpreted as biased science as it is postulating that the science suggests a different policy action, when in fact, you cannot derive a ‘should’ from an ‘is’ without the normative application of political values:

“I met somebody the other day and he said ‘[. . .]I only give the scientific facts, you know’ and I said ‘Oh, what was the last thing you did?’ and it was like some controversy [. . .] and he said ‘Oh yeah, that was like totally objective, I’m just spewing the facts’ and I go ‘but, why did you decide to get involved in that particular thing?’ ‘Why, you know, they just need to know the facts!’ ‘but how did you get to.’ . . . So clearly, it’s a subjective decision about what conversations, or what situations to get involved in – subjective, value based. What facts did you share with them – again, of all the billion different facts that you could give, right, which one did you share? You’re making a decision there, right?”

### Abuse of authority (A2)

Potential abuse of authority can occur when scientists engage in advocacy. By virtue of being ‘key holders’ to scientific knowledge, scientists are given a position of authority in their areas of expertise in comparison to a non-expert. An abuse of authority occurs when scientists are seen to be proselytizing on topics outside their expertise and present themselves as authorities on those matters. This is concerning because non-expert audiences lack the expert knowledge to judge what counts as being outside of scientist’s area of expertise, and therefore lend credence to what scientists say based on trusting that scientists only use their expert platform to discuss points that are within their area of expertise:

“What’s troubling is when people fluidly move across boundaries without declaring ‘I have expertise in this area, and not in that area’. Then it diminishes your credibility in areas where you do actually have scientifically grounded expertise.”

However, interviewees found it difficult to define expertise in practice. While specific criteria such as having a PhD can be used to distinguish between a layperson with substantial knowledge and an expert, distinguishing between areas of expertise is harder. Some interviewees proposed that the scientific training scientists received means they might be able to speak about neighbouring fields at a level that would still be classified as significantly more expert than a layperson, but less so than an expert in that exact field. However, deviating from the consensus view of that neighbouring community without declaring that they were voicing a dissenting or marginal view, could be seen as abuse of their position.

Abuse of authority may also occur when scientists wish to speak out as a citizen but are perceived as speaking out as an expert. Interviewees described tensions and uncertainty about whether they were able to operate in more than one role. Their experience was that even when they emphasized they were voicing views as a citizen and not an expert, audiences still viewed them as authoritative figures:

“That’s why you’re being interviewed, because you’re a scientist, not because you’re a random citizen they pick off the street. So, it’s a challenge. Because even if you do make that explicit [. . .] your words will have more weight. ‘He was talking as a citizen but it was interpreted as talking as a scientist with expertise, which is what got him into trouble’.”

This concern was mirrored in interviewees’ belief that the opinions of their peers significantly affect their careers and credibility. Some interviewees were deeply concerned about being accused of speaking out incorrectly about science, while others worried about being ‘tarred with the same brush’ if others spoke out:

“It’s better now, but I think the reason why people don’t want to talk out of science, in part, is not so much because they might make mistakes. It is the pressure of the research community, that they might make a mistake and people will hate them for that.”

Interviewees also shared their experience of being accused by the scientific community of engaging in unacceptable advocacy or presenting science in a way that could ‘fuel climate sceptics’. One interviewee shared:

“I remember two instances in particular where I was literally attacked, once publicly by a senior member of the research community. He didn’t mention me by name, but it was clear that he was talking about me and my mates. He said that we were just doing this for exposure and fame and that we didn’t know what we were talking about. It was really sort of shocking for me.”

Notably, many younger interviewees expressed a desire to engage in more advocacy, but were concerned about the potential risks, especially to their careers. Some expressed the willingness to not advocate until later in their careers when they had developed more of a reputation for creating sound science. One interviewee noted:

“Now, the other thing is it’s easier for an older scientist to be able to do these things because younger scientists are rightly worried about their careers, and they don’t get credit for this kind of action and often the opposite happens and they get penalised for it.”

### Defence of science (F1)

Interviewees highlighted the duties integral to being a responsible scientist included being a reliable source of information, ensuring accurate understanding when communicating to non-expert audiences, and defending science from misuse by other parties. In particular, interviewees expressed a strong commitment to speak out against false statements or misinterpretation of scientific findings, especially when policymakers misused scientific information to support specific policy positions:

“You have a duty to be honest, brutally honest. If you have evidence that someone’s claim is false, it should be your responsibility as a scientist to put your evidence out there.”

“My job is not to promote climate action, my job is to defend science. That is my number one.”

There was a strong sense from interviewees of having a duty to speak out, particularly when blatant mischaracterisations (such as deriving an ‘ought’ from an ‘is’) were being made about their own area of expertise, acknowledging greater responsibility to defend their ‘intellectual wheelhouse’:

“If it’s right within your intellectual wheelhouse, if it’s your daily scientific bread and butter, [. . .], and there are not many other people who are in a good position to rebut the incorrect claim or incorrect statement, then I think, you have some additional responsibilities.”

When these interviews were being conducted, there were several ‘Marches for Science’ in the United Kingdom and the United States, and increasing public debate about the role of experts in society and decision-making. Interviewees often defined ‘defence of science’ in this context as also including defending the authority of scientific knowledge and expertise. There was strong support for advocating for ‘science-based policy’. As mentioned in the section “Examples of perceived biased science in message and framing choices”, this is itself a form of policy advocacy, or at least can be perceived as such, when it is in response to a particular policy having been chosen. In this context, however, interviewees were describing the erasure of science from policy decision making completely:

“The president has, on the record, referred to climate change science as a hoax, as a conspiracy, and I have pushed back against that and I will continue to do that. So that’s lesson number one, you know – in the technical work that you do, there’s no point, as I see it, in being a scientist if you are unwilling to defend the technical work that you do.”

Similarly, the interviewees suggested that defending science in a perceived context of societal scepticism about the role of experts and the creation of scientific knowledge involves defending the scientific method as a means of generating universally valuable, politically unbiased information and discoveries.

### Duty as experts and citizens (F2)

A key concern in favour of advocacy was a duty to inform and warn the public about climate change. Many interviewees perceived a responsibility tied to their role as scientists:

“Because if you have relatively unique knowledge and you sit on it and it’s important that the world knows about it, what are people gonna think afterwards?”

Others often cited the public funding supporting their research as reason as to why they had a duty to make their research accessible to the public. For some, this duty extended to helping the public understand the implications of such research, and its relationship to policy options. Some also described how advocating for change, particularly in instances where scientific evidence indicates potential harm, was also part of their duty. They emphasized that remaining silent in the presence of knowledge capable of making a difference was deemed unethical:

“Almost nobody’s job is to save the world, but knowing something is going to have a negative consequence for other people, and to not say – that’s totally unethical.”

Others, however, had clear conceptions of their duty to communicate harm without stepping further into advocacy:

“As a scientist it’s a duty to give a public information talk, and I do, but then as uncomfortable as it is, it’s up to people as individuals to form decisions, then vote people into power.”

This duty to speak out, this time not in defence of science but out of concern for preventing harm, arose in two main ways: the duty of a concerned citizen, and the duty of an expert with the knowledge that can help prevent (further) harm:

“If we don’t speak out, I think it becomes a bit of an ethical issue [. . .] Like the doctor not sharing with the patient that you have cancer. Yeah? Or that you may have cancer and you should stop smoking. It’s exactly analogous.”

#### Examples of silence as advocacy

Interviewees expressed that simply avoiding advocacy by keeping silent is not a reliable way to be seen as remaining objective, and that silence can sometimes be seen as advocating for the other side:

“By my absence of advocacy, by my silence on these topics, I’ve been accused of being an advocate on the other side, implicitly.”

“Silence is complicity. Silence makes it easier to dismantle democratic norms and institutions, and to spread ignorance. And there’s no future in ignorance. Not for this country, not for this planet if we embrace ignorance.”

The silence of scientists in the context where others are misusing scientific research to support particular policy actions might be described by some in the scientific community as a dereliction of duty. Similarly, others clearly felt that silence in the face of climatic harm was also a dereliction of duty – both as a private citizen and as a scientist:

“Over a career there is nobody who doesn’t get millions of dollars in public funding to support what they’ve done. [. . .]I mean, do you think you deserve a million dollars ‘cos you’re cute? [. . .]. No, nobody deserves a million dollars [. . .] society’s paying you money. [. . .] I think that non-engagement is irresponsible and it’s selfish.”

## 5. Discussion

The debate over whether scientists should engage in climate advocacy has intensified recently, with more scientists participating in civil disobedience, particularly given the increasing urgency of the climate crisis (IPCC, 2023). This research aimed to uncover the fundamental concerns behind scientists’ views by conducting interviews with 47 climate scientists. Our findings reveal that the scientific community holds diverse views on advocacy and that individual scientists can hold multiple views too. These differing views often stem from varying judgments about how advocacy either upholds or threatens the integrity and credibility of science. We identify four fundamental concerns related to both epistemological and ontological dimensions: the risk of biased science, the potential abuse of authority, the duty to inform and warn the public, and the responsibility to defend science. Overall, our study clarifies how these concerns shape discussions about advocacy among climate scientists. By bringing these underlying concerns to light, our research contributes to a more nuanced, accurate, and productive discourse on the topic and helps prevent miscommunication or ‘talking past’ each other.

The current study has identified two fundamental concerns intrinsic to arguments in favour of the avoidance of advocacy, namely biased science and abuse of authority, and contributes a more in-depth comprehension of prior research on the subject (e.g. Boykoff and Oonk, 2020; Sharman and Howarth, 2017; van Eck, 2023). These concerns trace their origins to the ongoing debate surrounding the value-free ideal and, more broadly, the role of values in science. While the academic community has generally largely accepted that achieving entirely value-free science is impossible (with some exceptions, e.g. Büntgen, 2024), there is a need to revisit the discussion on what constitutes ‘acceptable’ value infusion (Douglas, 2009; Elliott, 2022; Nagel, 1961), especially as climate change increasingly threatens society (IPCC, 202). This discussion inevitably delves into considerations of the duties borne by scientists and when they are abusing their authority.

Defending the integrity of science emerged as a dual-faceted argument in our research, posited both in favour of ‘acceptable’ and ‘unacceptable’ advocacy. Some scientists argued that a certain type of advocacy is justifiable in order to uphold the credibility of climate science, while others maintained that any form of advocacy is impermissible to defend the independence of science. Both positions appeal to what the duties are for scientists when it comes to assessing the implications of their work, but are rooted in divergent epistemological reasoning and ontological understanding of the role of science and scientists in society. Recent work by Finnerty et al. (2024) supports this duality by showing how scientists’ perceptions of the relationship between science and activism significantly influences their stance on whether advocacy is compatible with maintaining scientific objectivity.

One argument emphasizes the need for science to be unbiased and that scientists have a duty to communicate the uncertainties inherent in science, given their better understanding of its complexities (Douglas, 2009). For instance, presenting science as more certain than it is can mislead decision-making. The other argument is whether scientists have a moral obligation to consider the societal implications of their research, given their insights into its impact on societal values. This perspective aligns with Brüggemann et al.’s (2020) description of post-normal science communication, where advocacy focuses on common goods like freedom of speech or sustainability rather than partisanship or self-interest. For future discussions about this topic, it is essential to clarify which fundamental concerns related to scientific integrity are being defended and how they align with different ontological views on the role of scientists in society.

While these discussions are well-established in the philosophy of science literature (e.g. Douglas, 2009; Elliott, 2022), they are currently being revisited in the climate science communication discipline (e.g. Kotcher et al., 2017; Post and Bienzeisler, 2024), given the increasing risks associated with climate change and the changing communication strategies of climate scientists. The communication of findings adds an important layer to this discussion. To illustrate, one challenge is that while scientific papers undergo a rigorous and systematic evaluation through peer review to filter out biased science (though its effectiveness can vary), public statements in science communication lack a similar formalized expert review. Future research could explore ways to prevent biased claims by scientists, while acknowledging that statements are inherently value-laden. Another challenge involves how scientists can clearly demarcate, if at all, when they communicate in their role of experts or as concerned citizens, while also being perceived as such by the public. More precisely, we need additional research to explore how value judgments and duties translate into science communication about climate change, including testing messages and frames (e.g. Cologna et al., 2022), for example about uncertainties and warnings (e.g. Budescu et al., 2014).

Beyond addressing our more fundamental question, our findings revealed that scientists fear judgement from the scientific community when communicating their findings to policymakers or the public, which is consistent with previous research (Boykoff and Oonk, 2020; van Eck, 2023). For some scientists, this fear manifested in self-censorship and silence. However, a novel finding of this research was that maintaining silence could also be perceived as a form of advocacy. The implications of such silence, as discussed by Painter et al. (2023) in the context of media coverage, extend to the scientific community as well, where silence might be seen as a strategic move, intentionally or unintentionally influencing public perception. The ramifications of such silence, both for the scientific enterprise and the future of the planet, warrant further elucidation. For instance, future research could empirically test the assumption whether silence is perceived as a form of assent.

Regarding practical implications, we recommend moving away from rigid demarcations between science and policy, science and activism, or scientists and citizens. Instead, we suggest integrating a value-based perspective from the philosophy of science to define what constitutes ‘acceptable’ and ‘unacceptable’ influences of values in climate science communication, based on the identified fundamental concerns (e.g. Cologna et al., 2022; van Eck et al., 2024). This perspective is often missing in current discussions about the roles of scientists in advocacy or activism (e.g. Büntgen, 2024; Dablander et al., 2024). For example, if a scientist values environmental protection and advocates for climate action, this does not necessarily imply that their science is biased; such behaviour might be categorized as ‘acceptable’. Conversely, conducting biased science is generally considered ‘unacceptable’, and public accusations of prejudiced science can lead to decreased trust in science. By incorporating this perspective, we encourage individual scientists, academic institutions and society at large to engage in discussions that more clearly delineate the acceptable influence of values in climate science communication. A clearer understanding of acceptable value infusion and the protections needed for scientific integrity will help define the roles and responsibilities of climate scientists.

This research is not without its limitations, which warrant careful consideration. First, the findings presented are solely based on the insights of the climate science community. Given that individuals in this discipline are not better equipped by training to think about these matters, it becomes imperative to incorporate perspectives from other domains, such as philosophers of science, the general public, or social scientists investigating climate change. Second, this research focused on the US and UK contexts. While these fundamental concerns may extend across national boundaries, it is important to consider the specific context when interpreting the results. Relationships between science and non-science vary by context (Gieryn, 1983); for instance, future research could explore climate science advocacy in the Global South, where communities face more direct impacts from climate change. Finally, this research identifies concerns and provides tools to navigate the debate, but refrains from making recommendations regarding the acceptability or non-acceptability of specific types of advocacy actions.

Overall, this research contributes novel insights to recent debates about climate advocacy by scientists, elucidating the foundational concerns that underpin their arguments. Each concern is inherently legitimate, adding complexity to the discourse. The existence of concerns on both ends of the debate underscores the challenging landscape scientists currently navigate. Yet, we argue that our identified fundamental concerns and philosophy of science perspective on the role of values in science can help guide discussions about the acceptability of climate advocacy by scientists.

## Supplemental Material

sj-docx-1-pus-10.1177_09636625251314164 – Supplemental material for Advocacy – defending science or destroying it? Interviews with 47 climate scientists about their fundamental concerns Supplemental material, sj-docx-1-pus-10.1177_09636625251314164 for Advocacy – defending science or destroying it? Interviews with 47 climate scientists about their fundamental concerns by Lydia Messling, Yuyao Lu and Christel W. van Eck in Public Understanding of Science
