# Supplementary material for: Advocacy – defending science or destroying it? Interviews with 47 climate scientists about their fundamental concerns
Source: Public Underst Sci. 2025 Feb 3;34(4):479–94. doi: 10.1177/09636625251314164 (PMC12038062; doi:10.1177/09636625251314164)
Supplement: sj-docx-1-pus-10.1177_09636625251314164 – Supplemental material for Advocacy – defending science or destroying it? Interviews with 47 climate scientists about their fundamental concerns [file sj-docx-1-pus-10.1177_09636625251314164.docx]

**Advocacy – defending science or destroying it? Interviews with 47 climate scientists about their fundamental concerns**

*Lydia Messling, Yuyao Lu, Christel W. van Eck*

**Supplemental Materials**

1. Appendix 1 List of interviewees (p.2)
2. Appendix II Codebook after first round of open coding (p.5)
3. Appendix III Final codebook (p.10)
4. Appendix IV Coded sample of the data (p.12)

**Appendix I List of interviewees**

*This is a list of interviewees that gave their consent to be named in this research. Their titles and affiliations are correct at the time of their interviews in 2018/ the publication of Lydia Messling’s Doctoral Thesis in 2020, which these interviews were conducted as part of.*

Prof. Michela Biasutti, Lamont Associate Research Professor, Lamont-Doherty Earth Observatory, Columbia University, USA.

Prof. Ken Caldeira, Senior Scientist at Carnegie Institution for Science, USA.

Prof. Jason Chilvers, Professor of Environment and Society, University of East Anglia, UK.

Prof. John R. Christy, Director of the Earth System Science Center, Distinguished Professor of Atmospheric Science, University of Alabama, USA.

Prof. Mat Collins, Joint Met Office Chair in Climate Change, University of Exeter, UK.

Dr. Judith Curry, President of Climate Forecast Applications Network, USA.

Dr. Thomas L. Delworth, Senior Scientist at GFDL/NOAA, Faculty Member in Atmospheric and Oceanic Science at Princeton University, USA.

Prof. Chris Field, Perry L. McCarty Director, Stanford Woods Institute for the Environment, Standard University, USA.

Prof. Dave Frame, Director of the New Zealand Climate Change Research Institute, University of Wellington, NZ.

Dr. Peter Gleick, President Emeritus of the Pacific Institute, USA.

Dr. Stephen M. Griffies, Lecturer in the Princeton University Atmospheric and Oceanic Sciences Program, Princeton, USA.

Dr. Luke Harrington, Postdoctoral Research Assistant, Environmental Change Institute, University of Oxford, UK.

Dr. Zeke Hausfather, Berkeley Earth, USA.

Prof. Isaac Held, Senior Research Scientist at GFDL/NOAA, USA.

Dr. Helene Hewitt, Science Fellow and Manager of the Ocean Modelling Group, Met Office, UK.

Prof. Mark Z. Jacobson, Professor of Civil and Environmental Engineering, Director of Atmosphere/Energy Program, Stanford University, USA.

Dr. Rachel James, Supernumerary Teaching Fellow, St John’s College, University of Oxford, UK.

Prof. Philip Jones, Professorial Fellow, Climatic Research Unit, School of Environmental Sciences, University of East Anglia, UK.

Prof. Manoj Joshi, Professor of Climate Dynamics, University of East Anglia, UK. Dr. Sarah Kapnick, Deputy Division Leader & Research Physical Scientist in Seasonal to Decadal Variability and Predictability Division, GFDL/NOAA, USA.

Dr. Till Kuhlbrodt, Model Configuration Manager for UKESM, National Centre for Atmospheric Science, UK.

Dr. Allegra N. LeGrande, Physical Research Scientist, NASA Goddard Institute for Space Studies, New York, USA.

Dr. Michael Mastrandrea, Director of Near Zero, Senior Research Associate at the Carnegie Institution for Science.

Prof. Denise Mauzerall, Department of Civil and Environmental Engineering and the Woodrow Wilson School of Public and International Affairs, Princeton University, USA.

Prof. Sonali McDermid, Assistant Professor in the Department of Environmental Studies, New York University, USA.

Mr Asher Minns, Executive Director of the Tyndall Centre for Climate Change Research, University of East Anglia, UK.

Dr. Doug McNeall, Climate Scientist and Statistician, Met Office Hadley Centre, UK.

Prof. Michael Oppenheimer, Albert G. Milbank Professor of Geosciences and International Affairs; Director of Centre for Policy Research on Energy and the Environment.

Dr. Friederike E. L. Otto, Acting Director of the Environmental Change Institute, and Associate Professor, Climate Research Programme, University of Oxford, UK.

Prof. Stephen W. Pacala, Frederick D. Petrie Professor in Ecology and Evolutionary Biology, Princeton University, USA.

Dr. Matt Palmer, Lead Scientist: Sea Level, Met Office Hadley Centre, UK.

Prof Amilcare M. Porporato, Department of Civil and Environmental Engineering and Princeton Environmental Institute, Princeton University, USA.

Prof. Corinne Le Quéré, Royal Society Research Professor of Climate Change Science at the University of East Anglia, UK.

Prof. Laure Resplandy, Assistant Professor of Geosciences and the Princeton Environmental Institute, Princeton University, USA.

Dr. Benjamin D. Santer, Program for Climate Model Diagnosis and Intercomparison, Lawrence Livermore National Laboratory, USA.

Dr. Gavin A. Schmidt, Director, NASA Goddard Institute for Space Studies, New York, USA. Prof Jason Smerdon, Lamont Research Professor, Lamont-Doherty Earth Observatory, Columbia University, USA.

Prof. David Stevens, Professor of Applied Mathematics, University of East Anglia, UK.

Dr Naomi Vaughan, Senior Lecturer in Climate Change, University of East Anglia, UK.

Prof. Gabriel Vecchi, Professor of Geosciences and at the Princeton Environmental Institute, Princeton University, USA.

Dr. Craig Wallace, Senior Research Associate, School of Environmental Sciences, University of East Anglia, UK.

Prof. Rachel Warren, Professor of Global Change and Environmental Biology, University of East Anglia, UK.

Dr. Michael White, Senior Editor, Climate Sciences - Nature Magazine, USA.

Mr Daniel Williams, Manager of Climate Knowledge Integration, Met Office, UK.

Dr. Phillip Williamson, Honorary Reader and NERC Science Coordinator, School of Environmental Sciences, University of East Anglia, UK.

*Two other interviewees wish to remain anonymous.*

**Appendix II Codebook after first round of open coding**

**1. DEFINING ADVOCACY**

1.1. What Advocacy is

1.1.1. Framing as Advocacy

1.2. What Advocacy is not

1.2.1. Manipulation

1.2.2. Support

1.2.3. SciComm

1.2.4. Advice

1.2.5. Stealth Advocacy

1.3. Unclear definition-understanding

1.4. Advocacy Spectrum

1.4.1. Areas

1.4.1.1. Non-engagement

1.4.1.2. Policy Advice

1.4.1.3. Action Advocacy

1.4.1.4. Specific Policy Advocacy

1.4.2. Boundaries

1.4.3. Mapping on Spectrum

1.4.3.1. Factors effecting mapping

1.4.4. Feedback

1.4.4.1. Positives

1.4.4.2. Criticisms

1.5. Silence as Advocacy

**2. ACCEPTABLE ADVOCACY**

2.1. Real Examples

2.1.1. Jim Hansen

2.1.1.1. Good

2.1.1.2. Bad

2.1.1.3. Mixed

2.1.2. Real Time Attribution Science

2.1.2.1. Good

2.1.2.2. Bad

2.1.2.3. Mixed

2.2. Theoretical Examples

2.2.1. Acceptable advocacy

2.2.2. Unacceptable advocacy

2.2.3. Uncertain about acceptability

2.3. Funding

2.3.1. Advocacy for

2.3.2. Tensions relating to

2.4. Identifying Audience

2.4.1. Acceptable for Policy Makers

2.4.2. Acceptable for General Public

2.4.3. Acceptable for Scientific Community

**3. SCIENTIFIC ATTRIBUTES**

3.1. Values

3.1.1. Individual

3.1.2. Shared

3.1.3. Conflicting values

3.1.4. Managing values

3.1.5. Communicating Values

3.2. Expertise

3.2.1. Defining-Identifying expertise

3.2.2. Limits of expertise

3.2.3. Boundaries of expertise

3.2.4. Abuse of expertise

3.2.5. Relationship with other characteristics

3.3. Consensus

3.3.1. Roles of consensus

3.3.2. Tensions of consensus

3.3.3. Pros/cons of consensus

3.3.4. Strength of consensus

3.3.5. Type of consensus

3.4. Authority

3.4.1. Defining-Identifying authority

3.4.2. Limits to authority

3.4.3. Threats to authority

3.4.4. Abuse of authority

3.5. Integrity

3.5.1. Defining-Identifying integrity

3.5.2. Importance-Role of Integrity

3.5.3. Type of integrity

3.5.4. Threats to integrity

3.6. Credibility

3.6.1. Defining-Identifying credibility

3.6.2. Importance-Role of credibility

3.6.3. Type of credibility

3.6.4. Threats to credibility

3.7. Humility

3.7.1. Defining-Identifying humility

3.7.2. Importance-Role of humility

3.7.3. Tensions of humility

3.8. Diversity

3.8.1. Defining-Identifying diversity

3.8.2. Importance-Role of diversity

3.8.3. Tensions of diversity

3.9. Interdisciplinarity

3.9.1. Defining interdisciplinarity

3.9.2. Importance of interdisciplinarity

3.9.3. Relationship to other characteristics

**4. RIGHTS & DUTIES**

4.1. Role-Duty of Science

4.1.1. In a democracy

4.1.1.1. 'Honest Broker'

4.1.1.2. 'Policy Adviser'

4.1.1.3. 'Pure Scientist'

4.1.1.4. 'Science Arbiter'

4.2. Role-Duty of Scientists

4.2.1. In a democracy

4.2.2. In scientific community

4.3. Role-Duty of Citizens

4.3.1. In a democracy

4.4. Freedom of Speech

4.4.1. Right to

4.4.2. Limitation of

**5. ETHICS OF COMMUNICATION**

5.1. Communication Frames

5.1.1. Choosing frames

5.1.2. Framing Uncertainties

5.1.3. Framing risk

5.2. Ethical Obligations

5.2.1. 'Sounding the alarm'

5.2.2. Being a reliable source

5.2.3. Correcting false statements

5.2.4. On being correctly understood

5.3. Communication Styles

5.3.1. Activism

5.3.2. Mediums of communication

5.4. Audience

5.4.1. Audience interaction

5.4.2. Audience identification

5.4.3. Audience influence

5.4.4. Responsibility for reaction

5.4.5. Forming dialogue

5.5. Motivations

**6. PRACTICAL METHODS**

6.1. Speaker

6.1.1. Expertise

6.1.1.1. Identifying and staying within area of expertise

6.1.1.2. Passing on to other experts

6.1.1.3. Acknowledging limits

6.1.1.4. Acknowledging transfer

6.1.2. Forming Consensus

6.1.2.1. For group agreement

6.1.2.2. For speaking as a group

6.1.2.3. With diversity

6.1.3. Motivations

6.1.3.1. How to recognise them

6.1.3.2. How to analyse them

6.2. Message

6.2.1. Recognising influences

6.2.2. Stating Values

6.2.2.1. Practical methods

6.2.2.2. Being reflexive

6.2.2.3. Being honest

6.2.2.4. Being personable

6.2.3. Forming content

6.2.4. Communicating uncertainties

6.2.5. SciComm

6.2.5.1. Good practice examples

6.3. Delivery

6.3.1. Communication Medium

6.4. Audience

6.4.1. Identifying the audience

6.5. Achieving Dialogue

6.5.1. Forming relationships

6.5.2. Cultivating trust

6.5.3. Listening well

6.6. Groundwork

6.6.1. Science Education for non-scientists

6.6.2. Training for Scientists

6.6.2.1. Philosophy of Science

6.6.2.2. Handling Journalists

6.6.2.3. Handling Sceptics

6.6.2.4. Reflexive practice

6.6.2.5. In how to speak to non-experts

**7. TENSIONS IN COMMUNICATING**

7.1. Communicating Uncertainties

7.2. Maintaining Trust. Integrity, Credibility

7.3. Complexity

7.4. Unknowns

7.5. Value-shoulds

7.6. Perception of Risk

7.7. Politically polarised problem

7.8. Competing Issues

7.9. Can't separate citizen from scientist

**8. OTHER KEY WORDS OR THINGS**

8.1. Views on Objectivity

8.2. Motivations or Rewards

8.3. Boundary Groups

8.4. In defence of Science

8.5. Career progress differences

8.6. Civil servants vs Higher Education

8.7. Non-related Advocacy

8.8. Reasons for non-engagement

8.9. Personality traits

8.10. IPCC

8.11. Applied Science

8.12. Dynamics of groups vs individuals

8.13. Publications & Peer Review

8.14. Country Differences

**Appendix III Final codebook**

This table presents the main codes from which the two key arguments—‘integrity of science’ and ‘role of scientists in society’—were derived. It includes codes used in the final selective coding phase. Note that some codes appear in more than one category due to thematic overlap. The numbers of the codes correspond with the themes of the interview guide.

|  | **Integrity of science (1)** | **Role of scientists in society (2)** |
| --- | --- | --- |
| **Advocacy threatens (A)** | **Biased science (A1)**  1.1.1. Framing as Advocacy 1.2.1. Manipulation  1.2.5. Stealth Advocacy 1.5. Silence as Advocacy  2.1.2. Real Time Attribution Science *(2.1.2.1. Good; 2.1.2.2. Bad; 2.1.2.3. Mixed)* 2.4.3. Acceptable for Scientific Community  3.1. Values *( 3.1.1. Individual; 3.1.2. Shared; 3.1.3. Conflicting values; 3.1.4. Managing values; 3.1.5. Communicating values)*  3.3 Consensus *(3.3.1. Roles of consensus; 3.3.2. Tensions of consensus; 3.3.3. Pros/cons of consensus; 3.3.4. Strength of consensus; 3.3.5. Type of consensus)*  3.5 Integrity *(3.5.1. Defining-Identifying integrity; 3.5.2. Importance-Role of Integrity; 3.5.3. Type of integrity; 3.5.4. Threats to integrity)*  3.6 Credibility *(3.6.1. Defining-Identifying credibility; 3.6.2. Importance-Role of credibility; 3.6.3. Type of credibility; 3.6.4. Threats to credibility)*  5.1 Communication Frames *(5.1.1. Choosing frames; 5.1.2 Framing uncertainties; 5.1.3. Framing risk);*  5.4 Audience *(5.4.1. Audience interaction; 5.4.2. Audience identification; 5.4.3. Audience influence; 5.4.4. Responsibility for reaction; 5.4.5. Forming dialogue)*  7.5 Value-shoulds  7.8 Competing issues  8.8. Reasons for non-engagement | **Abuse of authority/position (A2)**  1.1.1. Framing as Advocacy  1.2.1. Manipulation  1.2.3. SciComm  1.2.4. Advice 1.4.2 Boundaries 1.5. Silence as Advocacy  2.1.1. Jim Hansen *(2.1.1.1. Good; 2.1.1.2. Bad; 2.1.1.3. Mixed)* *2.3 Funding (2.3.1. Advocacy for; 2.3.2. Tensions relating to);*  2.4.3. Acceptable for Scientific Community  3.2. Expertise *(3.2.1. Defining-Identifying expertise; 3.2.2. Limits of expertise; 3.2.3. Boundaries of expertise; 3.2.4. Abuse of expertise; 3.2.5. Relationship with other characteristics)*  3.4 Authority *(3.4.1. Defining-Identifying authority; 3.4.2. Limits to authority; 3.4.3. Threats to authority; 3.4.4. Abuse of authority)*  3.5 Integrity *(3.5.1. Defining-Identifying integrity; 3.5.2. Importance-Role of integrity; 3.5.3. Type of integrity; 3.5.4. Threats to integrity)*  3.6 Credibility *(3.6.1. Defining-Identifying credibility; 3.6.2. Importance-Role of credibility; 3.6.3. Type of credibility; 3.6.4. Threats to credibility)*  4.1. Role-Duty of Science *(4.1.1. In a democracy; 4.1.1.1. ‘Honest Broker’; 4.1.1.2. ‘Policy Adviser’; 4.1.1.3. ‘Pure Scientist’; 4.1.1.4. ‘Science Arbiter’)*  4.2. Role-Duty of Scientists *(4.2.1. In a democracy; 4.2.2. In scientific community)*  5.1 Communication Frames *(5.1.1. Choosing frames; 5.1.2 Framing uncertainties; 5.1.3. Framing risk);*  5.3 Communication styles *(5.3.1. Activism; 5.3.2. Mediums of communication)*  5.4 Audience *(5.4.1. Audience interaction; 5.4.2. Audience identification; 5.4.3. Audience influence; 5.4.4. Responsibility for reaction; 5.4.5. Forming dialogue)*  7.2. Maintaining Trust, Integrity, Credibility  7.9. Can’t separate citizen from scientist  8.5. Career progress differences  8.8. Reasons for non-engagement |
| **Advocacy is needed (F)** | **Defence of science (F1)**  1.1.1. Framing as Advocacy  1.2.2. Support  1.4.2 Boundaries 1.5. Silence as Advocacy 2.3 Funding *(2.3.1. Advocacy for; 2.3.2. Tensions relating to);*  2.4.3. Acceptable for Scientific Community  5.1 Communication Frames *(5.1.1. Choosing frames; 5.1.2 Framing uncertainties; 5.1.3. Framing risk);*  5.2 Ethical Obligations *(5.2.1. ‘Sounding the alarm’; 5.2.2. Being a reliable source; 5.2.3. Correcting false statements; 5.2.4. On being correctly understood)*  5.4 Audience *(5.4.1. Audience interaction; 5.4.2. Audience identification; 5.4.3. Audience influence; 5.4.4. Responsibility for reaction; 5.4.5. Forming dialogue)*  7.1. Communicating Uncertainties  7.2. Maintaining Trust, Integrity, Credibility  7.7 Politically polarised problem  8.1. In defence of Science | **Moral obligation/right as a citizen (F2)**  1.2.2. Support  1.4.2 Boundaries 1.5. Silence as Advocacy 2.3 Funding *(2.3.1. Advocacy for; 2.3.2. Tensions relating to);*  2.4.3. Acceptable for Scientific Community 4.1. Role-Duty of Science *(4.1.1. In a democracy; 4.1.1.1. ‘Honest Broker’; 4.1.1.2. ‘Policy Adviser’; 4.1.1.3. ‘Pure Scientist’; 4.1.1.4. ‘Science Arbiter’)*  4.2. Role-Duty of Scientists *(4.2.1. In a democracy; 4.2.2. In scientific community)*  4.3. Role-Duty of Citizens *(4.3.1. In a democracy)*  4.4 Freedom of Speech *(4.4.1. Right to; 4.4.2. Limitation of)*  5.1 Communication Frames (5.1.1. Choosing frames; 5.1.2 Framing uncertainties; 5.1.3. Framing risk);  5.2 Ethical Obligations  5.3 Communication styles *(5.3.1. Activism; 5.3.2. Mediums of communication)*  5.4 Audience *(5.4.1. Audience interaction; 5.4.2. Audience identification; 5.4.3. Audience influence; 5.4.4. Responsibility for reaction; 5.4.5. Forming dialogue)*  7.1. Communicating Uncertainties  7.2. Maintaining Trust, Integrity, Credibility  7.7 Politically polarised problem  7.9. Can’t separate citizen from scientist  8.2. Motivations or Rewards  8.5. Career progress differences |

**Appendix IV Coded sample of the data**

Below is a sample of three pages from the node “7.9 Can’t separate citizen from scientist” which was used for understanding more about the ‘Role of scientists in society’ (both A2 and F2 categories). Interviewer speech is given in *italics*. Anonymised text within the quote is in bold. Brief commentary from the authors inference is provided in bold underneath some of the quotes.

**Sample of code “7.9 Can't separate citizen from scientist” (F2/A2)**

1. I mean that’s how I personally dealing with it, I mean of course, I see kind of like where you perhaps want to head with that because as a citizen, of course I could advocate much more clearly what I think, what type of climate change policy should be followed. But again, I cannot split this from me being a scientist, you know, if I would start to appear in the public sphere, then people would google my name and where I am from or where I currently live and then immediately they find out where I work and what I do, so it’s ridiculous to even try separating that – it simply can’t be done, you know…
2. [laughing] I mean everybody has got that problem, isn’t it? I mean, think of an NHS doctor, you know, who kind of like, in his or her daily experience sees how maybe currently the unit in the hospital she’s running, kind of like, is really suffering because there’s these staff shortages, you know. She could speak out in the public sphere but of course she could speak out as an NHS doctor even if she put a disclaimer in-front of every statement she makes; “I’m speaking as a citizen of this country not as the NHS doctor I am” but that disclaimer doesn’t really happen does it?
   **A perception that some form of disclaimer is needed in order for a scientist to speak out as a citizen – demonstrates a tension between fulfilling both one’s role as a citizen and as a scientist.**
3. It doesn’t. Because, I mean, you know, we’re all human, we all have points of view and, and editors are too.
4. I, I don’t have a bit of problem at all with that because I think as long as you’re separating... Now this is your role as... you know, your role and your right as an individual citizen. That’s fine. You’re not... You know, I’ve gone to, you know, climate protests in Washington. I don’t, you know, advertise myself as a climate scientist, I’m there as a citizen marching. It’s my right. But I don’t say my, my marching takes more precedence than your marching ’cos I’m, you know, in the field. No. I mean, so...
5. *So, if they were to do that as a citizen, would it still erode their credibility as a scientist?*
   If, if they were speaking as a citizen making ill-founded claims, I, I think so.
6. *Like do, do you think government labs would have a problem with the scientists doing what it is you do and speaking like from a personal story point of view?*
   No, I think they would not because if you preface it by saying this is my story, this is not a perspect... this is not an expression of **X**’s policy or advocacy, it is my personal story, they’re fine with that and they’ve... there’s been many statements...
   **Expressing the need to demarcate in order to respect the role of a scientist, here within governmental labs.**
7. Yeah. And that’s where I would not support this argument but that’s where the Rahmstorf’s get criticised. I, I think he’s a top notch scientist doing great stuff but taking his story and his message and saying, damn it, we’ve got to stop putting carbon into the planet because this is what I’m seeing, I’m seeing these signals, and I think that people see him... Knowing more than I have to know, he really believes that message so strongly, all of his science evidence is gonna be driven by that... shaped by that message.
   *So, he kind of loses his credibility to... or people question that he’s able to do his science strictly enough to the scientific method, that he wouldn’t have his values skewed or blindsided or...*
   Here’s it in a nutshell - that he places his heart before his head instead his head before his heart, and that’s where the academic community is very... I think it’s a problem because the heart and the head work together.
   **Suggestion that the role of the scientist is to ensure that scientific evidence is not driven by ‘heart’ messages/values, and yet that is also something that is hard to separate.**
8. You know, I, I feel that there’s a difference between the type of things that I’ve tried to do and the type of things where... that I’ve seen others do and I’m troubled by where... One is, is really trying to provide people with the information and then, when asked, you know, then you can say, listen, this is my opinion about how we should go forward. It’s informed by the information that I understand probably better than many people around the world but it’s also informed by my values and... that are a result of, you know, generations of culture that feed into me, my own personal experience, genes, who knows what, and so... So, there is a difference, right? So, it’s... If you qualify the things that are your opinion are your opinion, then maybe you’re on the cleaner side of advocacy. I don’t know.
   **Description of efforts to create a particular type of engagement that seeks to clearly communicate the personal values of the scientist to be considered in assessing their communication. Reference to a ‘clean side’ of advocacy, suggesting that there are advocacy roles/acts that are not permissible/dirty for scientists to engage in, and therefore should not be part of their role.**
9. *But then also remembering the fact that scientists are citizens with points of view and citizens’ voices should be listened to. So, the scientist is only ever purely just giving information to the policymaker and not at all expressing their policy preferences. Is that fair?*
   It’s probably not.
10. *So, controversial for climate change scientists to advocate specific policy or not?*
    For me, I think it is.
    *Yeah. And what, what is it...? So, this is what I’m trying to like really drill down on.*
    As a citizen I would. As a scientist, although I’m the same person, and I might have scientific arguments to say, oh, I think that’s a good idea or not but I don’t have all the policy arguments and social arguments that needs to be in the policy. But if people are willing to do it, okay, but I just don’t feel like I’m the person to do that. It would take all my time.
11. *And it’s asking a scientist to stop being a citizen.*
    Right? Well, right, and that’s exactly my argument. You cannot divorce the fact that you are both a scientist and a citizen.
12. *So some people I’ve spoken to talk about how they try and – when they speak out, to distinguish between when they are speaking out as themselves and when they are speaking out as a scientist -*
    Ahhh well – I would – I would phrase it slightly differently
    *OK?*
    I mean – you know – it’s uhh – I’s easy to say “look, here is what the science – the body of, the useful body of knowledge that everybody has come up with, including myself – says about this particular issue. Here is what I, as a citizen, think this means” Right? And so people do that a lot, and uh, I don’t – uh – I don’t think there’s any particular problem with that. But it’s not like, I’m not speaking - I mean, you know *[meaning the interviewer]*, you know enough of the um you know the science to tell because you speak knowledgably about the science without necessarily having contributed to anything, right? So there’s lots of people who know that as well, and they’re not speaking as scientists but they say exactly the same words as the scientists, right? Because they’re talking about [inaudible] science, right? So, err, so so, as a scientist as a citizen is not quite right. It’s “You’re always a citizen, you’re always a scientist”, - you are a scientist *[to the interviewer]* – Um, you’re talking about the science, versus, you know, your thinking, your your opinion about that. And people do distinguish these things. I mean, **X** like official policy is, err, you know, if you want to talk about science, talk, you know, share data, blah blah blah blah blah – you know, it’s our policy, just make it clear that you’re not speaking for **X**. But you’re not, you speak for yourself. And that, so I’m fine with that.
    **Reference to prescribed communication roles from an organisation – ensuring efforts are made to dissociate the communications of an individual from the organisation’s stance.**
13. Right? So, you’re not, you’re not, you’re a whole but you contain multitudes, right? And so part of that is, you know, deep knowledge of the sciences, part of that is your lived experience, part of that is humanitarian values or lack of, you know?
14. have opinions on what we should do policy-wise but in terms of me as a kind of... as a citizen as opposed to a scientist and so, you know, my dividing line, which is fuzzy, is kind of like I’m a scientist and I can tell you what happens if you put that in the atmosphere and, you know, the kind of implicit stance, you should not put that in the atmosphere. But then how would you not put that in the atmosphere or how do you reduce that? That’s kind of something that like I don’t have that much training on. And so sometimes people ask me like do you think nuclear power is a good idea. I’m like how would I know that? You know? Like I have, I have opinions as a citizen but, you know, you should not be asking, you know, a climate physicist for their opinions on like what a proper carbon tax is. And I think it’s like very appropriate for me to have opinions on those because I am a citizen and this is the world that I live in but I’m really wary of sort of representing myself as an expert.
    **Mention of appropriateness of statements being linked to one’s views as a citizen; that some opinions should not be expressed when being observed as operating in an expert role.**
15. And so I think… You know, we took a lot of hardened support from each other in doing that, you know, and then once we realised that this was all something that we wanted to do, it didn’t become easy but the commitment, you know, then… People got behind it very quickly once we all agreed that this wasn’t going to… On the surface we agreed that it wasn’t going to compromise our science. Inside we agreed that we’d all be brave enough to do this. Right? Like the conversation was actually masking something else that was going on in there that I think is human, like not… It’s not really necessarily just scientists, right? It’s just we had something to march for, finally. You know, not that… You know, individually you might care about other things but you don’t really… It’s unfortunate, right? You don’t necessarily understand the ramifications or empathise with them unless it impacts you and for once… for the first time in a long time the science as a whole in our perception was being impacted for funding, for… you know, for everything, legitimacy. And so I think that that was enough to personally impact a lot of people and it presented as an organisational thing but it was really a personal thing.
    **In reference to a March for Science. Talk of the tensions experienced when considering engaging in this activity explicitly as a scientist, in their role as a scientist. Also how motivations were both personally (as a citizen) and professionally motivated.**
16. So, yeah, I think it comes from a values place and, to be honest, I think the public is willing to accept scientists as citizens and scientists advocating and scientists, you know, pushing for certain policies and things so long as it’s understood where that’s coming from versus what they’re publishing on. So, yeah, I think that that’s really critical and, to be honest, to expect scientists to not have values or to not… Like I don’t think anybody would want that. I hope nobody wants that. Like I don’t think people want machines.
    *You want your scientists to think…*
    Yeah, you want your scientists to care.
    **Describing how operating in different roles as a citizen and a scientist is not possible in practice, nor actually desirable. Reference to scientists caring here – part of their role is not to be a “machine” but to engage in activities to show that they care – to deliberately allow personal values to influence their communications as a scientist.**
17. Yeah, exactly. That’s gonna bias everything. And we know… Like there are studies to show that this bias in peer review exists. So, this idea of scientists having to refrain from advocating a position because it might delegitimise and/or look, make their conclusions look biased or look unfounded is just ludicrous ‘cos you could make the most objective bang up, purely detach the study out there and if somebody doesn’t like the second name on your op-ed list, right, it’s gonna be… So, I think there’s this pretence that we really need to reckon with, the scientific community, before we start talking about advocacy and engagement. There’s this pretence of objectivism and this pretence of a purity, right, that is…
    *Value-free science.*
    …totally bogus.
18. My own view is not dissimilar to what you just stated. I think that to some degree science and the political realm are inseparable, both because the science that we do is influenced strongly by the priorities of the political realm and scientists always... I don’t mean all scientists. Many scientists have forever tried to influence the political... not only the political view of what science is good for, you know, to be, for instance, just for influencing funding decisions but also try to influence what issues policymakers thought about which had technical aspects, scientific aspects. This is true outside natural science. It’s true across expertise domains. So, you know, the idea... And then the separate idea that scientists or citizens is also... I feel strongly, should be an important component of how scientists think about their role. And I wanna be careful when I use the word should. A lot of this I don’t feel should be normative. It’s very individual.
    **Reference to scientists considering their role, and the relationship with their citizenship and engagement with politics and policy making.**
